# The Price per Prospective Consumer of Providing Therapist Training and Consultation in Seven Evidence-Based Treatments within a Large Public Behavioral Health System: An Example Cost-Analysis Metric

**DOI:** 10.3389/fpubh.2017.00356

**Published:** 2018-01-08

**Authors:** Kelsie H. Okamura, Courtney L. Benjamin Wolk, Christina D. Kang-Yi, Rebecca Stewart, Ronnie M. Rubin, Shawna Weaver, Arthur C. Evans, Zuleyha Cidav, Rinad S. Beidas, David S. Mandell

**Affiliations:** ^1^Department of Psychiatry, University of Pennsylvania, Philadelphia, PA, United States; ^2^State of Hawaii Child and Adolescent Mental Health Division, Honolulu, HI, United States; ^3^City of Philadelphia Department of Behavioral Health and Intellectual disAbility Services, Philadelphia, PA, United States; ^4^American Psychological Association, Washington, DC, United States

**Keywords:** evidence-based treatment, therapist, training and consultation, cost-analysis, population health

## Abstract

**Objective:**

Public-sector behavioral health systems seeking to implement evidence-based treatments (EBTs) may face challenges selecting EBTs given their limited resources. This study describes and illustrates one method to calculate cost related to training and consultation to assist system-level decisions about which EBTs to select.

**Methods:**

Training, consultation, and indirect labor costs were calculated for seven commonly implemented EBTs. Using extant literature, we then estimated the diagnoses and populations for which each EBT was indicated. Diagnostic and demographic information from Medicaid claims data were obtained from a large behavioral health payer organization and used to estimate the number of covered people with whom the EBT could be used and to calculate implementation-associated costs per consumer.

**Results:**

Findings suggest substantial cost to therapists and service systems related to EBT training and consultation. Training and consultation costs varied by EBT, from Dialectical Behavior Therapy at $238.07 to Cognitive Behavioral Therapy at $0.18 per potential consumer served. Total cost did not correspond with the number of prospective consumers served by an EBT.

**Conclusion:**

A cost-metric that accounts for the prospective recipients of a given EBT within a given population may provide insight into how systems should prioritize training efforts. Future policy should consider the financial burden of EBT implementation in relation to the context of the population being served and begin a dialog in creating incentives for EBT use.

## Introduction

In recent years, many efforts to improve mental health have focused on increasing the use of evidence-based treatments (EBTs) within public-sector service systems. Therapist training is a necessary—but not sufficient—implementation strategy to increase EBT use (1). For public-sector service systems, large-scale training of therapists is often the first or only EBT implementation strategy. A combination of experiential and active learning (e.g., didactic and case consultation) tends to produce the most favorable therapist behavior change over time (2, 3). As a result, many EBT developers and certifying organizations now require that therapists receive both didactic foundational training and ongoing case consultation to be “certified” in an EBT (e.g., PCIT International).^1^ Training and consultation require an investment on the part of therapists, their agencies, and, especially in publicly funded systems, the city or state agency that oversees payment for care. For example, therapists and organizations may incur initial direct costs like attending week-long trainings to first learn about the EBT and subsequently participate in weekly consultation calls for 6–12 months to ensure treatment fidelity. Therapists’ time required to participate often results in substantial cost to the agencies which they work (4–8). For example, Lang and Connell (6) estimated that an agency participating in a Trauma Focused-Cognitive Behavioral Therapy learning collaborative, which included agency-wide training and ongoing consultation, spent $89,575 in direct (e.g., training) and indirect (e.g., preparation hours) costs.

While public-sector service systems have typically used other strategies to select EBT, such as stakeholder feedback in combination with federal- and/or state-policy (9, 10), the breadth of the population served, and the associated costs should be important drivers of choice. Utilizing existing service system data is important for strategic decision-making and implementation tailored to the population (11). Information about the population served is needed to make decisions about where to invest their limited resources by understanding the extent to which an EBT provides diagnostic and demographic “coverage” within a service system (9). Costs associated with EBTs are often noted as significant barriers for implementation (12–14) and thus far the cost-analysis metrics that have been used to study implementation have not considered the population coverage relative to the implementation cost (4–8). A metric that considers the potential consumer served allows for population-based and data-informed decisions when selecting the right EBT. This metric can also inform cost-evaluative decisions on how applicable an EBT will be for each relevant consumer within the service system.

In this study, we introduce a strategy for calculating a cost per prospective consumer metric to determine the extent to which an EBT covers a given service system. To generate this metric, population data derived from that existing service system are needed; and within behavioral health, insurance claims (15), or practice-monitoring data tied to billing (9, 16) have predominantly been used. These large person-period datasets typically contain information regarding consumer age, gender, diagnoses, service utilization, and medication prescribed. This study was conducted to demonstrate the impact of therapist training and consultation costs in a large public behavioral health system and to describe a complimentary metric for system decision-making when selecting EBT for their population. First, training and consultation requirements for certification among seven EBTs were documented. Next, training, consultation, and indirect labor costs for each EBT were calculated. Finally, the total cost of training, consultation, and indirect labor for each EBT was divided across the number of potential consumers based on diagnostic and demographic information.

## Materials and Methods

### Evidence-Based Treatments

#### Identification of EBT

We identified EBTs for this study using registries created by the American Psychological Association (17), which rely on Chambless and Hollon (18) definitions of EBT. The APA’s Division 12 (Society for Clinical Psychology)^2^ and Division 53 (Society for Child and Adolescent Clinical Psychology)^3^ websites were consulted to determine EBTs that fit the criteria of (a) having an in-person training, (b) ongoing consultation period, and (c) a certifying body through which therapists can become “certified” in the particular EBT. Seven EBTs were identified through these websites: (a) Cognitive Behavioral Therapy/Cognitive Therapy (19), (b) Cognitive Processing Therapy (20), (c) Dialectical Behavior Therapy (21), (d) Parent–Child Interaction Therapy (22, 23), (e) Prolonged Exposure (24, 25), (f) Modular Approach to Therapy for Children with Anxiety, Depression, Trauma, and Conduct Problems (26), and (g) Trauma Focused-Cognitive Behavioral Therapy (27).

#### Training and Consultation Cost

The cost of training and consultation was determined using information from the certifying body for each EBT (see Table 1 for certifying bodies for each EBT). First, the certifying body’s website was referenced for certification requirements, upcoming trainings, and cost associated with training, consultation, and certification. When prices were not listed, we contacted the certifying body to solicit current prices and requirements for training and consultation to obtain certification. Revenue loss was defined as the total amount of therapist hours spent on training and consultation, as opposed to providing therapy (i.e., billable hours). Hourly wage for therapists, as determined by the US Bureau of Labor Statistics for Philadelphia,^4^ was established as $38.37 per hour.

**Table 1:** EBT training requirements.

EBT	Certifying body	Training hours	Consultation hours	Training length	Consultation notes
DBT	Behavioral Tech	40	50	12 months	12 months participation in one or more DBT consultation teams and current participation in DBT Team
PCIT	PCIT International	40	50	Case-based	Until two cases meet graduation criteria, applicant must remain in at least twice a month contact *via* real-time consultation (e.g., live, online, or telehealth observation, or video review)
PE	Center for the Treatment and Study of Anxiety, University of Pennsylvania	32	21	Case-based	Therapists receive one-one-one consultation (i.e., tape review) for two PE cases completed in a linear fashion, with some overlap allowable
CBT/CT	Academy of Cognitive Therapy Beck Institute	40	10	12 months	One year of clinical experience with at least 10 patients
CPT	Cognitive Processing Therapy Online	24	12	Time-based	Participation in 20 h of group consultation (with discussion of own clients) or a minimum of 12 h individual consultation
MATCH	PracticeWise	40	12	6 months	Receipt of at least 12 h of supervision or consultation over a 6-month period
TF-CBT	TF-CBT Therapist Certification Program	24	12	6–12 months	Twice a month for at least 6 months or once a month for 12 months

*EBT, evidence-based treatment; DBT, Dialectical Behavior Therapy; PCIT, Parent–Child Interaction Therapy; PE, Prolonged Exposure; CBT/CT, Cognitive Behavioral Therapy/Cognitive Therapy; CPT, Cognitive Processing Therapy; MATCH, Modular Approach to Therapy for Children with Anxiety, Depression, Trauma, or Conduct Problems; TF-CBT, Trauma Focused-Cognitive Behavior Therapy*.

#### Diagnostic and Age Applicability

To determine the population to which an EBT was applicable, diagnostic and age profiles were created for each EBT. We referenced APA’s Divisions 12 and 53 websites, the credentialing body’s website, and PracticeWise Evidence-based Services Database (28) to identify the studies used to establish each EBT’s efficacy. For example, Division 12’s website lists DBT as having Strong Research Support for Borderline Personality Disorder,^5^ with six efficacy trials used to determine that status. The Division 12 website also lists Strong Research Support for CBT/CT for Attention-Deficit/Hyperactivity Disorder, Insomnia, Binge Eating Disorder, Bipolar Disorders, Bulimia Nervosa, Depressive Disorders, Generalized Anxiety Disorder, Obsessive Compulsive Disorder, Social Phobia, Panic Disorder, and Schizophrenia; CPT for Posttraumatic Stress Disorder; and PE for Posttraumatic Stress Disorder. Efficacy trials for PCIT, MATCH, and TF-CBT were identified through comprehensive literature reviews cited by Division 53 (29–31) and the credentialing body’s website (PCIT International, PracticeWise, and TF-CBT National Therapist Certification Program, respectively).

Efficacy trials were coded by two independent raters (Kelsie H. Okamura and Courtney L. Benjamin Wolk) for diagnosis and age range used within each trial. Coders met to regularly resolve discrepancies, using clinical judgment and the conservative criteria of only including diagnoses that the EBT was intended to treat. Specifically for youth CBT, the PracticeWise Evidence-based Services Database (32, 33), a searchable database synthesizing more than 800 treatment studies for youth with psychiatric disorders, was referenced to determine a CBT youth diagnostic and age profile. The database was searched for CBT trials to identify diagnoses and age ranges that met well-established criteria proposed by Chambless and Hollon (18).

### Population-Based Data Source and Study Sample

Philadelphia County behavioral health Medicaid claims (*N* = 903,980) were used to identify a subset of consumers (*N* = 60,391) who received outpatient behavioral health services during November 2015 through October 2016. This 1-year time period was chosen because of the shift from ICD-9 and DSM-IV-TR diagnoses to ICD-10 and DSM-5 diagnoses. De-identified claims included age at the first claim, sex, race, psychiatric diagnosis, and behavioral health service use. Behavioral health services were categorized based on level of care codes and only claims reflective of outpatient therapy services were retained (i.e., assessment and medication management codes were excluded). The final sample included the consumers with two or more outpatient claims aggregated by ICD-10 diagnosis. Consumers may have been counted more than once across but not within ICD-10 diagnoses. This allowed for more consumer coverage and the ability to account for multiple psychiatric diagnoses. The University of Pennsylvania and the City of Philadelphia Department of Public Health Institutional Review Boards determined that this study was exempt from review due to the masking of identifiable information.

The final study sample included 897,064 claims representing 53,475 unique consumers. There were 6,916 duplicate consumers removed from analyses due to multiple claims being submitted for the same consumer for more than one diagnosis. In instances of multiple claims, the first claim per consumer was retained. Consumers were 53.4% female (*n* = 34,507) and averaged 29.91 (SD = 17.99) years of age. Race included African-American (42.7%, *n* = 27,573), Hispanic (37.8%, *n* = 24,339), White (15.6%, *n* = 10,061), and Other (3.9%, *n* = 2,531).

### Cost-Analysis Metric

The cost of therapist training, consultation, certification, and revenue loss were summed to calculate a total training and consultation cost for each EBT. This total training and consultation therapist cost was then divided by the number of consumers within Philadelphia County Medicaid claims who matched the EBT diagnostic and age profile. This formula resulted in an EBT training and consultation cost per potential consumer:

$$\frac{\text{TRAINING}+\text{CONSULTATION}+\text{CERTIFICATION}+\text{REVENUE LOSS}}{\text{NUMBER OF PROSPECTIVE CONSUMERS SERVED}}.$$

## Results

### Training and Consultation Requirements

Certifying bodies, training hours, consultation hours, training length, and specific criteria related to consultation are detailed in Table 1. Across EBTs, 2–5 days of in-person training were required for certification. TF-CBT and CPT both required online training in addition to the in-person training. Trainings were provided by certified trainers in each respective EBT, identified by the certifying body. Regarding consultation, DBT and PCIT required the most ongoing consultation (i.e., bimonthly contact for approximately a year), whereas CBT/CT required fewer hours (i.e., 1 year of clinical experience with 10 h of consultation). Live feedback in the form of tape review or telehealth observation was included in the consultation descriptions for PCIT and PE. Consultation hours typically spanned 6–12 months. MATCH and TF-CBT gave the option of meeting twice per month for 6 months or once per month for 12 months. PCIT and PE consultation were based on completion of two cases rather than a set time frame. CPT was similar in that it required 20 h of group or 12 h of individual consultation. Consultation was provided by a certified supervisor identified by the certifying body.

### Training and Consultation Cost

Training, consultation, certification, and revenue loss costs were summed to form a total cost in Table 2. EBT are rank ordered by their total cost, with DBT being the most expensive to TF-CBT being the least expensive. Training costs ranged from $585 for CPT to $4,900 for PCIT per therapist. However, consultation costs are included in the PCIT training cost. In addition to PCIT, MATCH and TF-CBT included the cost of consultation into their training cost. Stand-alone consultation prices ranged from $2,000 to $12,500, with consultation costs as either a set rate (i.e., $2,000 for CBT/CT consultation), per session rate (i.e., $185 for PE), or an hourly rate (i.e., $250 per hour for DBT, $200 per hour for CPT).

**Table 2:** Training and consultation costs per therapist.

EBT	Tuition	Consultation	Training and consultation	Certification	Revenue loss	Total
DBT	$2,485.00	$12,500.00	$14,985.00	$845.00	$3,453.30	$19,283.30
PCIT	$4,900.00	Included	$4,900.00	$225.00	$3,453.30	$8,578.30
PE	$1,500.00	$3,885.00	$5,385.00	Included	$2,033.61	$7,418.61
CBT/CT	$2,700.00	$2,000.00	$4,700.00	$450.00	$1,918.50	$7,068.50
CPT	$585.00	$2,000.00	$2,585.00	$250.00	$1,381.32	$4,216.32
MATCH	$1,900.00	Included	$1,900.00	$158.00	$1,995.24	$4,053.24
TF-CBT	$600.00	Included	$600.00	$250.00	$1,381.32	$2,231.32

*EBT, evidence-based treatment; DBT, Dialectical Behavior Therapy; PCIT, Parent–Child Interaction Therapy; PE, Prolonged Exposure; CBT/CT, Cognitive Behavioral Therapy/Cognitive Therapy; CPT, Cognitive Processing Therapy; MATCH, Modular Approach to Therapy for Children with Anxiety, Depression, Trauma, or Conduct Problems; TF-CBT, Trauma Focused-Cognitive Behavior Therapy*.

### Cost per Prospective Consumer

Prospective consumer costs were calculated by summing the total cost of training, consultation, certification, and revenue loss, and dividing that among the number of unique consumers fitting each EBT diagnostic and age profile. Table 3 details the total cost, age range in years, diagnoses, number of unique consumers fitting the diagnostic and age profile, and a cost per consumer (total cost/consumers) and is ordered by the per prospective consumer cost (most to least expensive). Cognitive Behavioral Therapy/Cognitive Therapy was the least expensive per consumer ($0.18) and covered the most prospective consumers (*n* = 39,586). In contrast, DBT was the most expensive per consumer ($238.07) and covered the fewest prospective consumers (*n* = 81).

**Table 3:** Evidence-based treatment (EBT) cost per consumer.

EBT	Total cost	Age range	Diagnoses	Potential consumers	Cost/consumer
Dialectical behavior therapy	$19,283.30	18–45	Borderline personality disorder	81	$238.07
Parent–child interaction therapy	$8,578.30	4–12	Adjustment disorders	2,672	$3.21
			Oppositional defiant disorder		
Cognitive processing therapy	$4,523.28	18+	Acute stress reaction	4,418	$1.02
			Adjustment disorders		
			Posttraumatic stress disorder		
			Reaction to severe stress		
Prolonged exposure	$7,418.61	13+	Adjustment disorders	4,926	$1.51
			Posttraumatic stress disorder		
			Reaction to severe stress		
Trauma focused-cognitive behavioral therapy	$2,231.32	3–17	Acute stress reaction	4,653	$0.48
			Adjustment disorders		
			Posttraumatic stress disorder		
			Reaction to severe stress		
Modular approach to therapy for children with anxiety, depression, trauma, and conduct problems	$4,053.24	7 to 13	Adjustment disorders	10,092	$0.40
			Anxiety disorders		
			Attention-deficit/hyperactivity disorders		
			Conduct disorder		
			Elimination disorders		
			Major depressive disorders (without psychosis)		
			Oppositional defiant disorder		
Cognitive behavioral therapy/cognitive therapy	$7,068.50	5+	Anxiety disorders	39,586	$0.18
			Attention-deficit/hyperactivity disorders		
			Bipolar disorders		
			Eating disorders		
			Major depressive disorders		
			Posttraumatic stress disorder schizophrenia		
			Substance use disorders		

## Discussion

The goal of this study was to develop a cost-analysis metric around the specific implementation strategy of EBT training and consultation while considering the population being served. This is particularly important given the financial pressures that large behavioral health services systems face to effectively implement EBT and manage tax-payer dollars and costs to the system, agencies, therapists, and consumers. Our study used seven common EBTs and compared training and consultation hours and prices and calculated per prospective consumer costs in a large behavioral health system. Training and consultation requirements and costs varied widely across EBT. Training and consultation costs ranged from $600 to $14,985 per therapist, and when considering certification fees and revenue loss from time spent in training rather than serving consumers, total costs ranged from $2,231.32 to $19,283.30. This represents a substantial investment to therapists, organizations, and systems. For some EBTs, consultation emerged as the most time-consuming and costly aspect, which is often emphasized as an important implementation strategy (2). Total cost did not correspond with the number of prospective consumers served by an EBT in our current behavioral health system sample. That is, the most expensive EBTs were not those that the most prospective consumers would benefit. This cost-analysis metric utilizing prospective consumer behavioral health outpatient claims appears to be a useful tool for large system decision-making in choosing EBT.

The costliest EBT to train (i.e., DBT) covered the fewest consumers in the system, likely because few consumers had a borderline personality disorder diagnosis. It is important to reiterate here that we used conservative diagnostic criteria for classifying which disorders a treatment was evidence-based for, and as such, may have excluded groups of consumers that may benefit from DBT (e.g., youth with suicidal ideation). We discuss this more in our limitations section as well as the cost-savings of having such a specialized EBT within a behavioral health system. Some of the less expensive EBTs provided greater consumer coverage. Systems considering which EBTs to invest in may wish to consider a tiered approach. That is, begin with (a) a generalist EBT (i.e., CBT/CT and MATCH) and then consider adding on (b) trauma focused (i.e., TF-CBT, PE, or CPT), and (c) other specialty EBT (i.e., PCIT and DBT) depending on the prospective consumers served. The proposed cost-analysis metric may be particularly useful for systems seeking to understand the financial impact of specialty EBT (34). While most costly in our study, if a specialty EBT like DBT aligns well with system priorities, such as reducing inpatient hospitalization rates, residential treatment utilization, or other out of home placement, it may make the additional investment worthwhile. Furthermore, it may be beneficial for systems to create a ratio of therapists trained to prospective consumers served to inform future training efforts. This tiered approach also has implications for research which is beginning to suggest that attitudes (35) and knowledge (36) vary by practices and EBT, suggesting that our field’s conceptualization of EBT as all-encompassing may be misguided. Moreover, treatment developers may wish to consider building modularity and tiered decision-making into interventions to increase applicability to a broader range of consumers. A tiered approach to choosing and conceptualizing EBT may facilitate decisions about which EBTs to compare and study within effectiveness and implementation studies (e.g., comparing two generalist type EBTs rather than a specialty EBT and generalist EBT).

In-person didactic training and ongoing consultation were required across all seven EBTs for certification. The typical time period for in-person training was 1 week (40 h); however, CPT and TF-CBT required only 2 days (24 h) in-person training with completion of an additional online course as a pre-requisite for certification. Reviews of empirical studies on training have concluded that didactic training alone does not produce change in therapist behavior and should be combined with ongoing feedback and consultation (2, 3). However, it is unclear from the literature the extent to which didactic trainings need to be delivered in-person and the requisite amount of training hours to attain competency. Our findings suggest an emerging standard of 40 h for didactic training. From a system’s perspective, taking cohorts of service-delivering therapists offline for a week may be perceived as both costly and detrimental to consumers receiving services. However, if multiple systems begin to adopt this convention of training and consultation as requirements for employment and credentialing as well as enhance outpatient rates to absorb some of those costs, they may be more acceptable and feasible to provider agencies.

Ongoing consultation requirements also place considerable demand on the therapist and system. In this study, consultation requirements were observed to vary even more than didactic training requirements. For example, CPT, MATCH, and TF-CBT required 12 h of supervision across varying time frames (e.g., 6–12 months, see Table 1), whereas DBT and PCIT required a year of ongoing consultation with bimonthly attendance. Research has suggested that the purpose of ongoing consultation is to give the trainee the opportunity to apply the skills learned in didactics with sufficient supervision and support (37, 38). Typically, consultation entails ongoing case-review, which may or may not take the form of reviewing session recordings or live feedback. Indeed, only PCIT and PE included live or taped feedback as a part of their consultation model. Consistent with didactic training, the frequency, and depth of consultation needed to fully achieve competency has not been established and this may impact cost. For example, consultation with review of session recordings is more time-consuming than case-based discussions. Furthermore, research on training and sustainability has noted that even when therapists are comprehensively trained and supervised in EBT they do not use EBT frequently in their practice (2). Determining the optimal duration and format for didactic training and consultation should be an implementation science priority. For public-sector service systems, there are likely many considerations when deciding which EBT(s) to invest in including time, cost, policy, and population-based characteristics. For example, should a service system first choose an EBT that requires less training and consultation (e.g., MATCH) over one that requires a longer training and consultation time frame (e.g., DBT) to increase EBT capacity quickly? The answer to this question is beyond the scope of this study. However, initial findings suggest that the variation between EBTs is substantial enough to warrant further attention.

The results of this study should be considered within the context of several limitations. First, our study used administrative Medicaid claims data, which may not be reflective of the entire service-seeking population (e.g., private insurance covered consumers or population prevalence within the community). Furthermore, several studies have suggested that Medicaid claims data may not be diagnostically accurate (39–41). However, studies have demonstrated that the agreement of Medicaid claims diagnoses to clinical data is around 85% (39, 40) suggesting that the inaccuracy of claims may be related to an under-identification of disorders rather than inaccuracy of diagnosis. Also related to diagnosis, some of the efficacy trials that we coded to create age and diagnostic profiles included multiple psychiatric diagnoses, which may suggest that the corresponding EBT would be appropriate for both the intended and comorbid conditions. In these instances, we took a conservative approach and only considered the diagnoses for which an EBT primarily targeted. For example, a trial of DBT for individuals with borderline personality disorder and cooccurring substance use was coded as effective for adults with a diagnosis of borderline personality disorder but not for individuals with a primary substance use disorder diagnosis. Future studies may wish to examine broader diagnostic categories (e.g., depressive disorders versus major depressive disorder) or behavioral codes (e.g., suicidality), which may represent a more inclusive approach. In addition, replication using national epidemiological data with standardized diagnostic assessments [e.g., Ref. (42, 43)] would circumvent concerns about diagnostic accuracy and provide additional insight into the proportion nationally that might benefit from specific EBTs. It is also important to note that this cost-analysis metric, while not statistically or methodologically difficult to apply, does require some expertise in using claims data. Therefore, public-sector service systems will need administrators, analysts, or external research/evaluation partners to apply the cost-analysis metric to claims datasets.

We examined the costs associated with specific implementation strategies (i.e., training and consultation) without considering the effectiveness of the intervention itself (i.e., cost-effectiveness analysis, especially in the case of DBT). Raghavan (44) has noted that estimating implementation costs is different from cost-effectiveness as it is influenced by the entity (e.g., system, agency, and therapist) to which the cost is associated as well as the strategy, EBT, and setting (45). Our goal was to understand the direct and indirect costs at the population level that may be associated with the implementation strategy of EBT training and consultation in a large public behavioral health system. One important caveat was that training and consultation costs were calculated at the individual therapist level, which may not parallel costs for system-wide trainings in the community (46). Often, partnerships and contracts are executed to train and provide consultation for large cohorts of therapists within the system versus using a cost per therapist model (47). In addition, indirect costs were calculated based on therapist wage loss during training and consultation (and not revenue loss to the provider agency), without accounting for other contributing activities to sustaining the EBT including supervision, non-billable preparation hours, and travel time. Again, our focus was on the implementation strategy of training and consultation and is consistent with other studies that have evaluated a discrete amount of time as a part of the indirect implementation cost (44). Furthermore, Beidas et al. (12) have demonstrated that high turnover often affects the fiscal landscape of EBP implementation and our study did not account for loss on investment or the extent to which a therapist needed to stay within the system for a good return on investment. System policy makers, administrators, and researchers will need to collaboratively set standards for training requirements and cost and conduct cost-effectiveness studies that are linked to consumer outcomes.

Despite these limitations, this study proposes a methodology for considering which EBT to choose within a large behavioral health system. We propose a tiered approach to selecting EBT, allowing our cost-analysis metric, stakeholder feedback, and system priorities to influence the selection. Our cost calculations may also serve as a basis for policy around incentivizing the use of EBT (1), especially in the early stages of implementation when the system and agency can expect a loss in revenue due to therapist productivity and agency revenue. For example, Timmer and Urquiza (48) described a demonstration project in Los Angeles County Department of Mental Health that reimbursed agencies for lost productivity hours during an initial training initiative. While some systems have mandated the use of EBT (49, 50), few systems have begun to incentivize the use of EBT (i.e., Chester County, PA, USA; City of Philadelphia Department of Behavioral Health and Intellectual disAbility Services). Understanding the effectiveness of mandates and incentives in therapist utilization and consumer receipt of EBT as well as improved clinical outcomes will be the next era of implementation research, and developing pragmatic cost-analysis metrics will enable large systems to make decisions about which EBT to adopt for whom. Moreover, developing methods and testing them within and across large systems of care will enhance implementation science and generalizability of findings in health services research.

## Author Contributions

KO was responsible for all aspects of this manuscript, from conceptualization to writing. CW and DM provided consultation in conceptualization and editing. CK-Y performed data analysis of Medicaid claims data. ZC provided consultation in health economics. RS, RB, RR, SW, and AE provided additional feedback and editing of the manuscript.

## Conflict of Interest Statement

No authors declare any personal, professional, or financial relationships that could potentially be construed as a conflict of interest. The reviewer AB and handling Editor declared their shared affiliation.

## Footnotes

^1^http://www.pcit.org/.

^2^http://www.div12.org/psychological-treatments/.

^3^http://effectivechildtherapy.org/.

^4^https://www.bls.gov/bls/blswage.htm.

^5^http://www.div12.org/psychological-treatments/disorders/borderline-personality-disorder/dialectical-behavior-therapy-for-borderline-personality-disorder/.
